# Hyperbaric Oxygen Environment Can Enhance Brain Activity and Multitasking Performance

**DOI:** 10.3389/fnint.2017.00025

**Published:** 2017-09-27

**Authors:** Dor Vadas, Leonid Kalichman, Amir Hadanny, Shai Efrati

**Affiliations:** ^1^The Israeli Rehabilitation Center for Stroke and Brain Injury, Rehovot, Israel; ^2^Department of Physical Therapy, Faculty of Health Sciences, Recanati School for Community Health Professions, Ben-Gurion University of the Negev, BeerSheva, Israel; ^3^Sagol Center for Hyperbaric Medicine and Research, Asaf Harofeh Medical Center, Zerifin, Israel; ^4^Sackler School of Medicine, Tel Aviv University, Tel Aviv, Israel; ^5^Galilee Faculty of Medicine, Bar Ilan University, Ramat Gan, Israel; ^6^Sagol School of Neuroscience, Tel Aviv University, Tel Aviv, Israel

**Keywords:** HBOT, hyperbaric oxygenation, dual tasking, oxygen limitation, enhancing brain activity, NCT03126669, 213/13

## Abstract

**Background:** The Brain uses 20% of the total oxygen supply consumed by the entire body. Even though, <10% of the brain is active at any given time, it utilizes almost all the oxygen delivered. In order to perform complex tasks or more than one task (multitasking), the oxygen supply is shifted from one brain region to another, via blood perfusion modulation. The aim of the present study was to evaluate whether a hyperbaric oxygen (HBO) environment, with increased oxygen supply to the brain, will enhance the performance of complex and/or multiple activities.

**Methods:** A prospective, double-blind randomized control, crossover trial including 22 healthy volunteers. Participants were asked to perform a cognitive task, a motor task and a simultaneous cognitive-motor task (multitasking). Participants were randomized to perform the tasks in two environments: (a) normobaric air (1 ATA 21% oxygen) (b) HBO (2 ATA 100% oxygen). Two weeks later participants were crossed to the alternative environment. Blinding of the normobaric environment was achieved in the same chamber with masks on while hyperbaric sensation was simulated by increasing pressure in the first minute and gradually decreasing to normobaric environment prior to tasks performance.

**Results:** Compared to the performance at normobaric conditions, both cognitive and motor single tasks scores were significantly enhanced by HBO environment (*p* < 0.001 for both). Multitasking performance was also significantly enhanced in HBO environment (*p* = 0.006 for the cognitive part and *p* = 0.02 for the motor part).

**Conclusions:** The improvement in performance of both single and multi-tasking while in an HBO environment supports the hypothesis which according to, oxygen is indeed a rate limiting factor for brain activity. Hyperbaric oxygenation can serve as an environment for brain performance. Further studies are needed to evaluate the optimal oxygen levels for maximal brain performance.

## Introduction

The brain is the body's largest consumer of oxygen, utilizing roughly 20% of the total oxygen and consuming 25–30% of the total glucose (Lennie, 2003). Even though <10% of the brain's maximal capacity is active at every given time, the brain utilizes almost all delivered oxygen (Sokoloff et al., 1955). In order to perform different tasks or more than one task at a time (multitasking), the oxygen supply is shifted from one region of the brain to another via blood perfusion modulation (Lennie, 2003). These perfusion changes can be easily visualized by functional magnetic resonance tomography (fMRI) technology (Tombu et al., 2011). Multiple studies have demonstrated that our ability to perform complex activities decreases under oxygen depleted environments (Shukitt-Hale et al., 1998; Lieberman et al., 2005; Malle et al., 2013). However, the possible effect on brain performance by a single hyperbaric oxygen (HBO) environmental exposure has not been studied in humans.

Brain performance is highly sensitive to any decrease in oxygen supply. A reduction of the plasma oxygen pressure to 65 mmHg will impair the brain's ability to perform complex tasks, at 55 mmHg short-term memory will be impaired, while at <35 mmHg consciousness will be lost (Zauner, 2002). The effects of a hypobaric environment (decreased oxygen level) in individual motoric and cognitive performances were studied at high altitudes. At high altitudes or other oxygen depleted environments, cognitive and motor performances are impaired while performing relatively simple tasks (Shukitt-Hale et al., 1998; West, 2002, 2003; Mortazavi et al., 2003; Lieberman et al., 2005; Malle et al., 2013). On the other hand, elevation of oxygen levels, even at normobaric conditions, was found to facilitate cognition by decreasing the response time in the elderly (Choi et al., 2013). In a randomized control trial where memory consolidation (as a measure for cognition) was evaluated, increased oxygen supply at normobaric conditions (sea level) to healthy young participants, word memorization was more efficient compared to control group (Moss and Scholey, 1996).

While multitasking at a normal environment (normal air at sea level), oxygen is required in multiple brain regions simultaneously. The relative deficiency of oxygen may explain the decrease in processing speed, accuracy and other neuro-cognitive performances (Spelke et al., 1976; Han and Marois, 2013). It is assumed that conscious attention to two different actions performed at the same time is possible only if the tasks are coordinated into a single, higher-order activity (Spelke et al., 1976) or that at least one of the activities is being done “automatically” without conscious awareness (Spelke et al., 1976; Han and Marois, 2013). Donohue et al. have found that the ability to perform more than one task is limited even in individuals who are very experienced in one of the given tasks (Donohue et al., 2012).

**The aim** of the present study was to evaluate whether an HBO environment, with increased oxygen supply to the brain, will enable better performance of complex and/or multiple activities in healthy individuals.

## Materials and methods

A prospective, double blind randomized control, crossover trial was performed at the Sagol Center for Hyperbaric Medicine and Research, Assaf-Harofeh Medical Center, Israel. This study was carried out in accordance with the recommendations of Assaf-Harofeh medical center institutional review board with written informed consent from all subjects. All subjects gave written informed consent in accordance with the declaration of Helsinki. The protocol was approved by the Center's institutional review board.

### Participants

The study included 22 healthy volunteers, aged 20 years or older with a minimum of 12 years of formal education. Patients were excluded if they had any inner ear pathologies, lung disease, or any mental or physical limitations of being exposed to a hyperbaric chamber environment.

### Protocol and end-points

**Figure 1:**
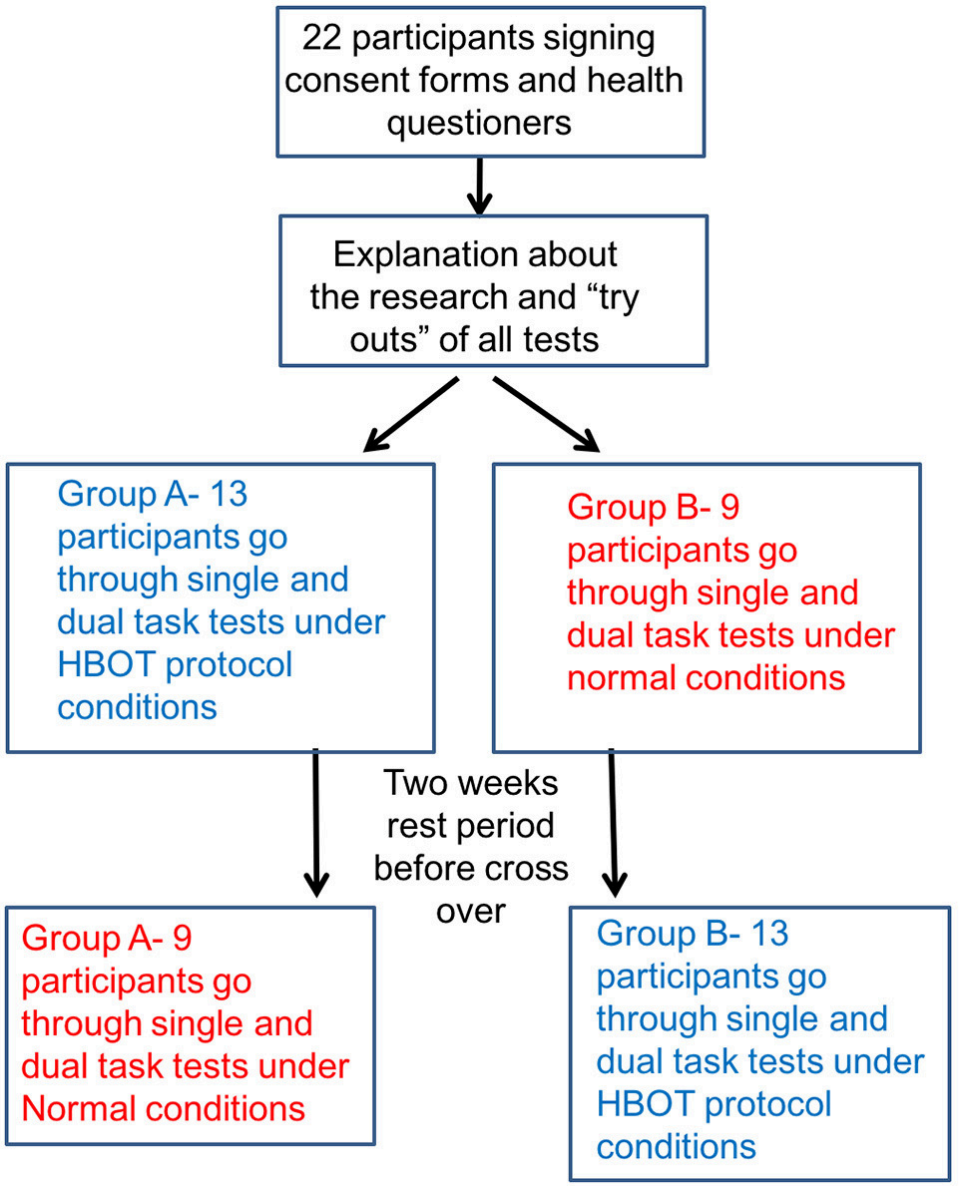
Study protocol diagram.

Participants were randomly assigned to be evaluated in one of the two environments: normal room air in normal pressure (control) and HBO (intervention). After a 2-week wash-out period, participants were crossed to the alternative environment (Figure 1).

During each session, participants were seated in the chamber with masks on. In the intervention session, the pressure in the chamber was increased to 2 ATA, and the participants breathed 100% oxygen for 45 min. In the control session, in order to manipulate the participants, the chamber pressure was increased for less than a minute so they felt some pressure in the ears, and a flow of 21% oxygen was used for the masks. The tests/tasks were initiated 30 min after the masks were on within the chamber.

Cognitive performance was evaluated by a symbol-digit test (example in Appendix 1), and the motor performance was evaluated by the units of beans that were transferred from a plate to a cup (One of the tasks in the motor assessment scale test-MASS, Appendix 2). The dual tasking was a combination of the cognitive and the motor tests which were done simultaneously.

### Cognitive/attention symbol-digit modality test (SDMT)

The symbol digit modalities test (SDMT, Appendix 1) is a test used to assess divided attention, visual scanning, tracking, and motor speed (Strauss et al., 2006). In this test, participants are presented with series of symbols, each indicates a different number. While they are repeatedly presented with those different symbols they are asked to write the adequate number next to each symbol (the Appendix includes an example of the task recoding). The test was found to be reliable in four different forms in healthy and cognitive impaired conditions (McCaffrey, 1988; Hinton Bayre and Geffen, 2005; Dickinson et al., 2007; Paramenter, 2007). Three different forms of the SDMT were used in each evaluation session. The first was a try-out for the test, ensuring that the task was understood. The second tested the ability to perform an individual task. The third form was used in combination of the motor task for examining dual task performance. Standard administration procedures were followed as indicated in the test manual (Smith, 1973). The score of the test is the number of correct substitutions during a 90-s interval.

### Motor task: transferring of beans

Motor performance was evaluated by the motor assessment scale test (MAS, Appendix 2)—transferring individual beans between two tea cups an arm's length away. The MAS test was found to be reliable for assessing advance hand-motor functioning in post stroke patients (Carr, 1985; Pool and Whiteny, 1988). One of the advantages of this task is that during its performance the participant is not required to have full vision focus on the hands, allowing the participant to do other tasks. In addition, the test can be easily performed under the conditions inside a hyperbaric chamber. The MAS score is based on the number of beans that were successfully transferred in the given time (90 s).

### Statistical analysis

Statistical analysis was done using SPSS software (version 22.0). Continuous data are expressed by mean ± STD (standard deviation) and compared by an independent *t*-test for inter-group comparison and by a paired *t*-test for intra-group comparison. *P*-values < 0.05 were considered statistically significant. All randomly allocated patients were included in the safety analysis and those that went through all the assessments were included in the efficacy analyses.

## Results

Twenty-four healthy participants signed an informed consent and were included in the study. Two patients were excluded: One did not complete the crossed evaluation as required by study protocol and the other could not adjust to the hyperbaric conditions. Accordingly, a total of 22 healthy volunteers were included in the final analysis (11 females and 11 males, age 22–68 years (mean 42 ± 13 years) with 12–30 education years at the time of the study (mean 16 ± 4 years). Baseline participant characteristics are summarized in Table 1. The results of the cognitive and the motor tests are summarized in Table 2.

**Table 1:** Patient baseline characteristics.

		**All participants**
Number of participants		22
Gender	Female	11
	Male	11
Age		42 ± 13
Years of education		16.3 ± 4

**Table 2:** Cognitive and motor task scores.

	**General**		
	**Room air**	**Hyperbaric oxygen**	***P*-value**
Single task Cognitive	40.2 ± 9.8	43.9 ± 11.6	*P* < 0.001
Dual task Cognitive	35.2 ± 10.8	38.7 ± 11.7	*P* = 0.006
Single task Motor	83.8 ± 9.3	89.3 ± 11.5	*P* < 0.001
Dual task Motor	51.4 ± 14.7	56 ± 14.2	*P* = 0.029

A significant performance decline was observed in all tests scores when performed dual tasks compared to single tasks in both normobaric and HBO environments (*p* < 0.001 for both).

**Figure 2:**
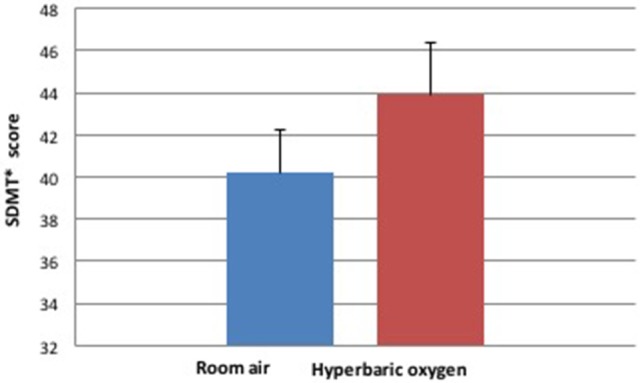
Cognitive single task during hyperbaric oxygen compared to normal environments. ^*^SDMT—Symbol Digit Modality Test.

**Figure 3:**
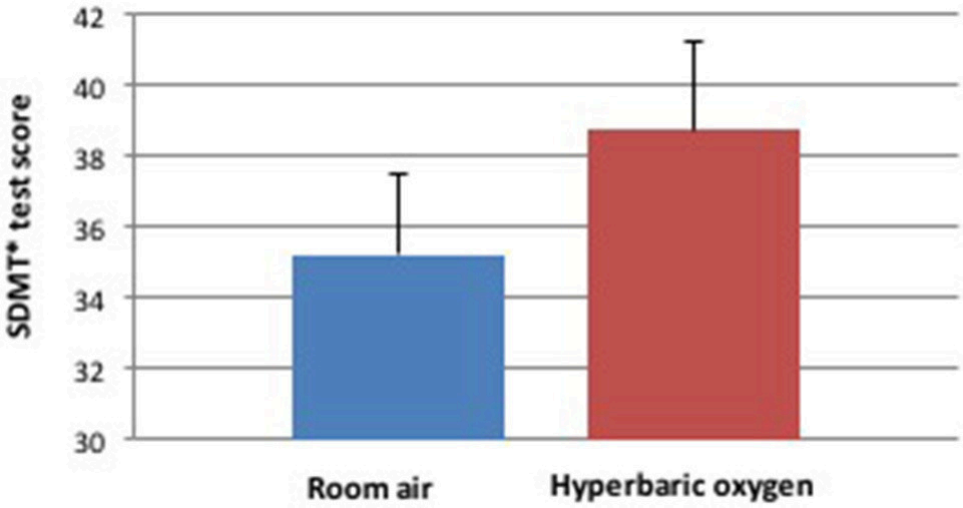
Cognitive score during dual task during hyperbaric oxygen compared to normal environments. ^*^SDMT—Symbol Digit Modality Test.

**Figure 4:**
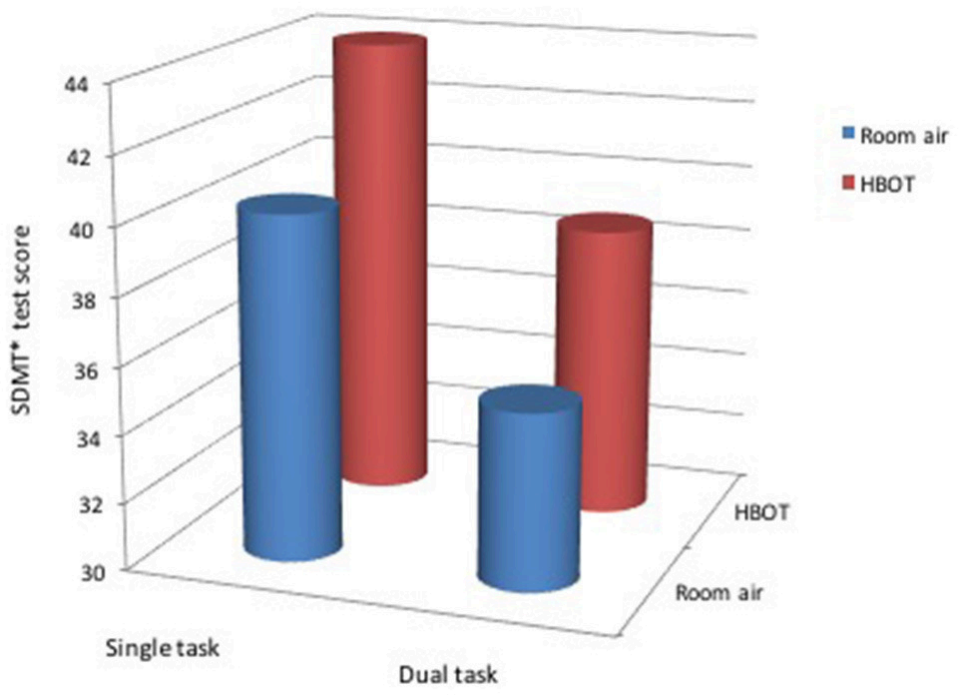
General cognitive score during hyperbaric oxygen compared to normal environment. ^*^SDMT—Symbol Digit Modality Test.

The SDMT cognitive task was considered the primary task and the motor task was considered the distraction when we analyzed the dual task performance at the two different environments. SDMT scores, the number of correct answers minus the incorrect answers in the test, were significantly increased when performed under the HBO environment compared to the normobaric environment (Table 2, Figures 2–4). The improvement in SDMT in the HBO environment was significant when tested either as a single (43.9 ± 11.6 vs. 40.2 ± 9.8, *p* < 0.001) (Figure 2) or as a dual task (38.7 ± 11.7 vs. 35.2 ± 10.8, *p* = 0.006) (Figure 3).

Motor task scores were also significantly increased when performed under the HBO environment compared to the normobaric environment. The improvement in the motor task results, or the number of bean units that were transferred from the plate to the cup, under the HBO environment was significantly higher either as a single (83.8 ± 9.3 vs. 89.3 ± 11.5 *p* < 0.001) or as a dual task (51.4 ± 14.7 vs. 56 ± 14.2 *p* = 0.029).

## Discussion

The study's findings indicate that even for healthy individuals, oxygen at normal conditions is a limiting factor for brain activity. The ability to perform cognitive and/or motor tasks as a single or a combined task (multitask) was evaluated at a normal air/oxygen environment and at HBO, a hyperbaric oxygen enriched environment. Increasing oxygenation using a HBO environment significantly enhanced both cognitive and motor performance. Significant improvements were found for both single tasks as well as simultaneous multiple tasks.

Many studies have confirmed that environments low in oxygen levels have a negative effect on performing single and multiple tasks (Shukitt-Hale et al., 1998; Lieberman et al., 2005). A study performed by Yu et al. evaluated the effect of repeated exposure to HBO treatment for healthy individuals (Yu et al., 2015). Their study demonstrates that repeated daily HBO sessions, 5 days per week, 80 min with 100% at 2 ATA, can enhance memory performance. The improved memory correlated with enhanced functional connectivity in the left hippocampus, right inferior frontal and lingual gyri as demonstrated by fMRI analysis (Yu et al., 2015). In the current study, the immediate effect of a single HBO environment exposure was evaluated in healthy volunteers. Since it is a single exposure, the immediate effect on neuro-cognitive performance cannot be related to the neuroplasticity effects of HBO but rather to a limiting factor preventing the brain to function at a higher capacity. This finding means that oxygen is indeed a limiting factor for brain performance at standard conditions in healthy human beings.

The minimal effective dosages of pressure and oxygen concentration are still unknown, and future studies are needed to test this issue by evaluating the optimal, case-specific dose response curves (Efrati and Ben-Jacob, 2014). There is a huge variability in HBO dosages (1.3–2.4 ATA) used in previous studies aimed to induce neuroplasticity in post stroke and TBI patients (Boussi-Gross et al., 2013; Efrati and Ben-Jacob, 2014; Tal et al., 2015; Hadanny and Efrati, 2016). Oxygen is not a drug and because it is mainly metabolized in the mitochondria, its pharmacodynamics varies greatly from patient to patient. Thus, no simple dose-response curve has been defined so far. Emphasizing the latter, there are many case reports illustrating the significant effect of relatively low increases in air pressure (Golding et al., 1960; Austin, 1998). For example, the Dead Sea (altitude 402 m below sea level, 1.05 ATM) can serve as a good model for a relatively “low” hyperbaric environment. The beneficial effect of this slight increase in air partial pressure is well-known and was studied and evaluated in different populations (Kramer et al., 1998; Falk et al., 2006; Goldbart et al., 2007). With respect to the current study, the aim was to confirm that oxygen is indeed a rate limiting factor for enhancing the activity and not to investigate the minimal effective dosage oxygen. For that purpose, we chose to use 100% oxygen at 2 ATA as the challenge dose. Now that we have demonstrated that indeed oxygen is a limiting factor, further studies are needed to evaluate the dose-response curve related to enhancing brain/cognitive performance.

In today's modern life, there is an increased need for multitasking, which is unfortunately limited (Carrier et al., 2009). The inability to perform well while multitasking could have severe and even life threatening consequences, as was found in emergency room care physicians (Chisholm et al., 2000) and in military drone pilots (Shanker and Richtel, 2011). Considering that an oxygen enriched environment could enhance performance, improve multitasking and decision making, the use of this environment could have a significant impact for those who needs it. However, before being used for large scale populations, the minimal and maximal effective dosage should be evaluated.

In addition to immediately enhancing brain/cognitive functions, there is growing convincing evidence that HBO therapy can revitalize chronically damage brain tissue in patients suffering from chronic neuro-cognitive impairment due to TBI, stroke or anoxic brain damage even years after the acute insult (Boussi-Gross et al., 2013; Efrati et al., 2013; Efrati and Ben-Jacob, 2014; Hadanny et al., 2015; Tal et al., 2015; Hadanny and Efrati, 2016). As detailed above, brain metabolism reaches its upper limit of oxygen consumption even at normal healthy conditions, which makes it dependent on cerebral blood flow (CBF) for its oxygen supply. After brain insults, when the CBF is compromised, there is a further decrease in oxygen delivery to the injured brain tissue and oxygen becomes a limiting factor for brain recovery (Hadanny and Efrati, 2015). Consequently, achieving higher tissue oxygen delivery by using higher paO_2_ is crucial for maintaining the sufficient oxygenation needed for the damaged brain tissue (Hadanny and Efrati, 2015). Clinical studies published in recent years present convincing evidences that HBO therapy (HBOT) can assist in brain repair (Boussi-Gross et al., 2013, 2015; Efrati and Ben-Jacob, 2014). In addition to delivering sufficient oxygen to the brain for tissue repair, HBOT might initiate cellular and vascular repair mechanisms and improve cerebral vascular flow (Efrati and Ben-Jacob, 2014). At the cellular level, HBOT can improve mitochondrial function (in both neurons and glial cells), improve blood-brain barrier and inflammatory reactions, reduce apoptosis, alleviate oxidative stress, increase levels of neutrophils and nitric oxide, and upregulate axon guidance agents (Efrati and Ben-Jacob, 2014). Moreover, the effects of HBOT on neurons can be mediated indirectly by glial cells, including astrocytes (Efrati and Ben-Jacob, 2014). HBOT may also promote neurogenesis of the endogenous neural stem cells (Efrati and Ben-Jacob, 2014). At the vascular level, HBOT was found to have a role in initiating and/or facilitating angiogenesis and cell proliferation needed for axonal regeneration (Efrati and Ben-Jacob, 2014).

Another potential effect of HBOT may be its possible contribution to perception. Perception provides meaning for sensation. Mendez-Balbuena et al, has shown that by providing audio tactile stimulation, the sensory experience (perception) of vision, could be expended (Mendez-Balbuena et al., 2015). It might be possible that enhanced brain activity by HBOT may also increase sensory perception. However, it was not directly evaluated in the current study and it could be an interesting goal for additional studies.

The current study has several challenges and potential limitations. One important limitation relates to the test re-test learning effect due to the crossover design. Every participant has performed the tests twice under both conditions in separate sessions. To overcome this limitation, participants were randomly divided into two groups in a way that part of the participants started under the HBO environment and the other part started with the sham environment (normobaric with room air). Accordingly, the two groups are almost matched for their learning effect. The other challenge is related to generating the control intervention that would mimic hyperbaric environment where participants can sense the increased pressure in their ears. To overcome this challenge, the chamber pressure was increased and then gradually decreased during the control session so that the participants felt some pressure in their ears, and a flow of 21% oxygen was used for the masks. The tests/tasking were initiated 30 min after the masks were on within the chamber and the pressure at that time was already reduced back to sea level during the placebo session.

## Conclusion

The improvement in performance of both single and multitasking while in a HBO environment supports the hypothesis that oxygen is indeed a rate limiting factor for brain activity. Hyperbaric oxygenation can serve as an environment for enhancing brain performance. Such a brain enhancing environment can be of significant importance when many skills are becoming more and more dependent on enhanced cognitive functions and multitasking. Further studies are needed to evaluate the optimal oxygen-performance relation for maximal brain performance.

## Author contributions

All authors listed have made a substantial, direct and intellectual contribution to the work, and approved it for publication.

### Conflict of interest statement

The authors declare that the research was conducted in the absence of any commercial or financial relationships that could be construed as a potential conflict of interest.
