# Supplementary material for: Hyperbaric Oxygen Environment Can Enhance Brain Activity and Multitasking Performance
Source: Front Integr Neurosci. 2017 Sep 27;11:25. doi: 10.3389/fnint.2017.00025 (PMC5623811; doi:10.3389/fnint.2017.00025)
Supplement: Supplementary file 1 [file DataSheet1.docx]

**Appendix**

1. Example of Symbol digit modality test form:


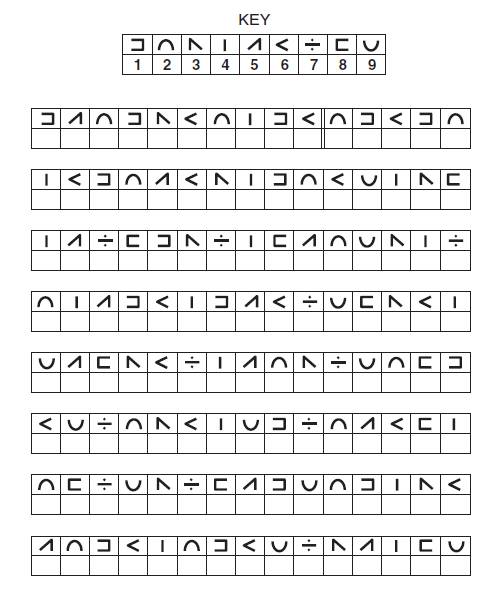


2. Motor Assessment Scale test:

**MOTOR ASSESSMENT SCALE**

Agency:__________________________ PID #: _______________ Date: _____________ CPT #:____________

Patient Name: _____________________________________ Therapist: __________________________________

*If the patient cannot complete any part of a section score a zero (0) for that section. There are 9 sections in all.*

**Upper Arm Function**

1. Supine: Therapist places affected arm in 90 degrees shoulder flexion and holds elbow in extension – hand toward ceiling.

The patient protracts the affected shoulder actively.

2. Supine: Therapist places affected arm in above position. The patient must maintain the position for 2 seconds with some

external rotation and with the elbow in at least 20 degrees of full extension.

3. Supine: Patient assumes above position and brings hand to forehead and extends the arm again. (flexion & extension of

elbow) Therapist may assist with supination of forearm.

4. Sitting: Therapist places affected arm in 90 degrees of forward flexion. Patient must hold the affected arm in position for 2

seconds with some shoulder external rotation and forearm supination. No excessive shoulder elevation or pronation.

5. Sitting: Patient lifts affected arm to 90 degrees forward flexion - holds it there for 10 seconds and then lowers it with some

shoulder external rotation and forearm supination. No pronation.

6. Standing: Have patient’s affected arm abducted to 90 degrees with palm flat against wall. Patient must maintain arm position

while turning body toward the wall.

**Hand Movements**

1. Sitting at a table (Wrist Extension): Affected forearm resting on table. Place cylindrical object in palm of patient’s hand.

Patient asked to lift object off table by extending the wrist – no elbow flexion allowed.

2. Sitting at a table (Radial Deviation of Wrist): Therapist should place forearm with ulnar side on table in mid-pronation /

supination position. Thumb in line with forearm and wrist in extension. Fingers around cylindrical object. Patient is asked to

lift hand off table. No wrist flexion or extension.

3. Sitting (Pronation / Supination): Affected arm on table with elbow unsupported at side. Patient asked to supinate and pronate

forearm (¾ range acceptable).

4. Place a 5 inch ball on the table so that the patient has to reach forward with arms extended to reach it. Have the patient reach

forward with shoulders protracted, elbows extended, wrist in neutral or extended, pick up the ball with both hands and put it

back down in the same spot.

5. Have the patient pick up a polystyrene cup with their affected hand and put it on the table on the other side of their body

without any alteration to the cup.

6. Continuous opposition of thumb to each finger 14 x in 10 seconds. Each finger in turn taps the thumb, starting with the index

finger. Do not allow thumb to slide from one finger to the other or go backwards.

**Advanced Hand Activities**

1. Have the patient reach forward to pick up the top of a pen with their affected hand, bring the affected arm back to their side

and put the pen cap down in front of them.

2. Place 8 jellybeans, (beans), in a teacup an arm’s length away on the affected side. Place another teacup an arm’s length away on

the intact side. Have the patient pick up one jellybean with their affected hand and place the jellybean in the cup on the intact

side.

3. Draw a vertical line on a piece of paper. Have the patient draw horizontal lines to touch the vertical line. The goal is 10 lines

in 20 seconds with at least 5 lines stopping at the vertical.

4. Have the patient pick up a pen/pencil with their affected hand, hold the pen as for writing, and position it without assistance

and make rapid consecutive dots (not strokes) on a sheet of paper. Goal: at least 2 dots a second for 5 seconds.

5. Have the patient take a dessert spoon of liquid to their mouth with their affected hand without lowering the head toward the

spoon or spilling.

6. Have the patient hold a comb and comb the back of their head with the affected arm in abduction and external rotation,

forearm in supination.
